# Sugar Beet (*Beta vulgaris*) Guard Cells Responses to Salinity Stress: A Proteomic Analysis

**DOI:** 10.3390/ijms21072331

**Published:** 2020-03-27

**Authors:** Fatemeh Rasouli, Ali Kiani-Pouya, Leiting Li, Heng Zhang, Zhonghua Chen, Rainer Hedrich, Richard Wilson, Sergey Shabala

**Affiliations:** 1 Tasmanian Institute of Agriculture, College of Science and Engineering, University of Tasmania, 7001 Hobart, Australia; Fatemeh.Rasouli@utas.edu.au (F.R.); ali.kianipouya@utas.edu.au (A.K.-P.); 2 Shanghai Centre for Plant Stress Biology and CAS Centre for Excellence in Molecular Plant Sciences, Chinese Academy of Sciences, Shanghai 201602, China; ltli@psc.ac.cn (L.L.); zhangheng@sibs.ac.cn (H.Z.); 3 School of Science and Health, Hawkesbury Institute for the Environment, Western Sydney University, 2747 Penrith, Australia; Z.Chen@westernsydney.edu.au; 4 Institute for Molecular Plant Physiology and Biophysics, University Wuerzburg, 97082 Wuerzburg, Germany; hedrich@botanik.uni-wuerzburg.de; 5 Central Science Laboratory, University of Tasmania, 7001 Hobart, Australia; 6 International Research Centre for Environmental Membrane Biology, Foshan University, Foshan 528000, China

**Keywords:** guard cells, stomata, sugar beet, salt stress, proteomic

## Abstract

Soil salinity is a major environmental constraint affecting crop growth and threatening global food security. Plants adapt to salinity by optimizing the performance of stomata. Stomata are formed by two guard cells (GCs) that are morphologically and functionally distinct from the other leaf cells. These microscopic sphincters inserted into the wax-covered epidermis of the shoot balance CO_2_ intake for photosynthetic carbon gain and concomitant water loss. In order to better understand the molecular mechanisms underlying stomatal function under saline conditions, we used proteomics approach to study isolated GCs from the salt-tolerant sugar beet species. Of the 2088 proteins identified in sugar beet GCs, 82 were differentially regulated by salt treatment. According to bioinformatics analysis (GO enrichment analysis and protein classification), these proteins were involved in lipid metabolism, cell wall modification, ATP biosynthesis, and signaling. Among the significant differentially abundant proteins, several proteins classified as “stress proteins” were upregulated, including non-specific lipid transfer protein, chaperone proteins, heat shock proteins, inorganic pyrophosphatase 2, responsible for energized vacuole membrane for ion transportation. Moreover, several antioxidant enzymes (peroxide, superoxidase dismutase) were highly upregulated. Furthermore, cell wall proteins detected in GCs provided some evidence that GC walls were more flexible in response to salt stress. Proteins such as L-ascorbate oxidase that were constitutively high under both control and high salinity conditions may contribute to the ability of sugar beet GCs to adapt to salinity by mitigating salinity-induced oxidative stress.

## 1. Introduction

Plant leaves are composed of diverse types of cells such as mesophyll, vascular tissues, epidermis, and guard cells (GCs), each of which play different roles in plant growth, development, and response to environmental stimuli. GCs are specialized paired cells in the leaf epidermis forming stomata for gas exchange between plant inside and surrounding atmosphere and are morphologically distinct from the other leaf cells. They adjust the stomatal aperture by changing turgor pressure in response to internal or environmental cues. When stomata are fully open, CO_2_ passes through to be absorbed by mesophyll cells for photosynthesis and water transpires and escapes from the leaf surface. However, stomata pores can be closed or adjusted to only be partially open to save water in response to some stresses caused, for example, by reduced water availability. Among environmental factors, salinity can have a serious impact on plant yield. With the reported rate of soil salinization 3 ha/min [1], salinity becomes a major challenge worldwide [2]. Moreover, the current trends in population dynamics, urbanization, and climate change will exacerbate the process of land salinization. Consequently, understanding stomatal control of plant water transport and CO_2_ assimilation under saline conditions is becoming increasingly important [3]. Currently, studies on stomatal operation under salt stress are mainly limited to physiological measurements such as stomatal conductance and stomatal apertures at the whole-plant level. Only a very limited number of studies have focused on transcriptional and metabolomic changes in GCs per se, largely because of challenges associated with obtaining a sufficient number of isolated and purified cells for the experiment [4].

Proteomic analysis has been applied in single cell-type studies as an effective method for detecting key proteins and pathways in biological systems [5,6]. While tissue-level studies can be informative, cell type-specific information is lost in tissues composed of multiple cell types such as whole leaf. In contrast, isolated populations of specific cell types have the potential to unravel novel biological processes underlying specific protein functions in the specialized differentiated cells. A good example is the study by Barkla et al. [7], demonstrating differential abundance of proteins involved in ion and water homeostasis between leaf lamina and epidermal bladder cells (EBC) in *Mesembryanthemum crystallinum* (ice plant) under saline conditions. In the current study, GC-enriched epidermal fragments were isolated from the leaves of sugar beet plants grown under control and saline conditions to study the proteome profile of GC-specific responses to salt stress. Few studies have used proteomics to investigate the protein profile of GCs and, to the best of our knowledge, there is no report of proteomic analysis of GCs under saline conditions. In the earliest proteomics study on GCs, Zhao et al. [4] used protoplasts isolated from Arabidopsis epidermis by enzymatic digestion of epidermal fragments, followed by a combination of 2-D gel and LC-MALDI MudPIT methods. Subsequently, Zhu et al. [8] identified guard cell specific proteins in *Brassica napus* that were related to transcription, cell structure, signaling, and energy metabolism. In contrast to GCs, proteins involved in photosynthesis and starch synthesis were enriched in mesophyll cells. Proteomics has also been used to analyze the response of GCs to environmental cues, including light, CO_2_, and ABA [9,10]. For example, proteins and metabolites associated with fatty acid metabolism, redox regulation, and starch /sucrose metabolism were identified in *Brassica napus* GCs in response to low CO_2_ [9]. This finding shows that the knowledge obtained from proteomic experiments on GCs can boost our understanding of specific cellular events and key proteins involved in stress conditions and provide information about molecular networks underlying the stomatal functions in different conditions.

For the current stomatal study, we chose sugar beet (*Beta vulgaris ssp. vulgaris*) because it is a species of the Amaranthaceae family with high-salinity stress tolerance. A wild ancestor of sugar beet is sea beet (*Beta vulgaris ssp*. *maritima*) which is well-adapted to high saline environments of coastal areas. Sugar beet has inherited salt-resistance characteristics and is classified as a salt-tolerant halophilic plant [11]. Although this plant has been domesticated relatively recently, it has not been adversely affected by the domestication process as it has maintained its resistance to high salinity levels [12]. Therefore, this plant could be a good resource to explore salt tolerance mechanisms and to identify proteins and genes involved in salt tolerance [13]. Currently, proteomic studies on salt tolerance in sugar beet have been limited to tissues such as shoot, root, and leaf [2,14,15], with no studies on GCs reported to date. In this study, the GCs isolated for proteomic analysis were validated with microscopic observation, viability tests and comparing guard cell-specific proteins in mesophyll and guard cell samples. We report the overall protein profile of the isolated GCs in addition to proteins differentially abundant under saline conditions. Functional classification and Gene Ontology (GO) enrichment analysis of the GC proteome and differentially abundant proteins were used to identify characteristic, dominant proteins, the most enriched biological processes in whole guard cell proteome and those proteins affected by salinity.

## 2. Results

### 2.1. Stomatal Traits and Conductance in Sugar Beet 

**Figure 1:**
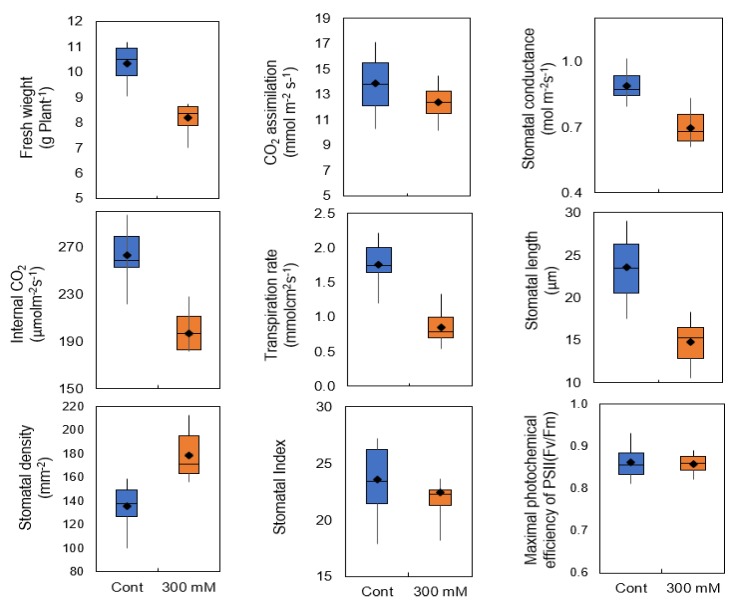
Fresh weight, CO_2_ assimilation, stomatal conductance, and stomatal length, density in the sugar beet exposed to 300 mM NaCl for three weeks. Error bars correspond to the SE of at least 5-15 biologically independent measurements.

**Figure 2:**
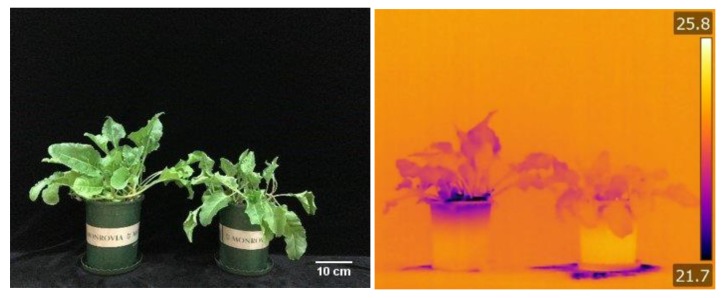
Sugar beet grown for three weeks under control and 300 mM NaCl, normal imaging shows the effect of salinity stress on sugar beet growth (**A**) and thermal imaging indicates higher rates of transpirational cooling in plants under control conditions (B). One (of 5) typical images are shown for each panel.

**Figure 3:**
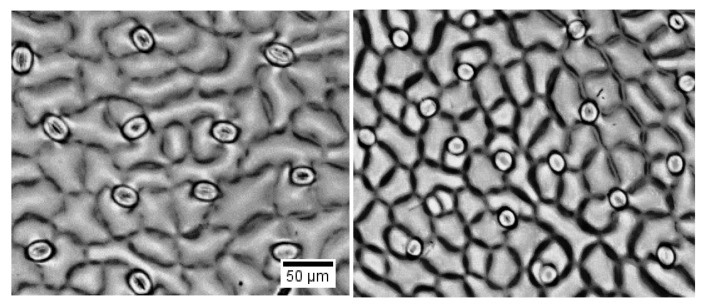
Stomatal density and size in the abaxial leaf surface of sugar beet plants grown under control (A) and saline (300 mM NaCl for 3 weeks) conditions. One (of 8-12) typical images is shown for each panel.

We recognized sugar beet growth was stimulated at 100 mM NaCl with a small reduction in fresh weight at 200 mM NaCl (Supplemental Figure S1). In the present study, treatment with 300 mM NaCl for three weeks resulted in 21% and 11% decline in fresh weight and net CO_2_ assimilation, respectively (Figure 1 and Figure 2), which can be attributed to the limited stomatal conductance and consequently lower concentration of CO_2_ in the intercellular spaces of the leaf. We observed that stomatal length was reduced by salinity in sugar beet, while at the same time stomata density was increased (Figure 1 and Figure 3).

More importantly, the stomatal index (the ratio between the number of stomata and epidermal cells per unit area) remained unchanged, suggesting that stomatal development and differentiation was not influenced by salinity in sugar beet and suggests that the increased number of stomata is more likely to be attributed to decline in total leaf area due to osmotic stress. The maximal photochemical efficiency of PSII (Fv/Fm) was 0.87 in control treatment and did not change significantly at 300 mM NaCl treatment suggesting that PSII reaction centers may not be the main target of salinity stress.

Given that reduced transpiration increases leaf temperature, infrared thermal imaging was used to compare the temperature of sugar beet leaves under control and salinity stress (Figure 2). The temperature of the salt-treated leaves was higher than those of the control leaves, suggesting a reduced rate of transpiration in salt-treated plants.

### 2.2. Comparison of Ground State Mesophyll and Guard Cells Proteome

**Figure 4:**
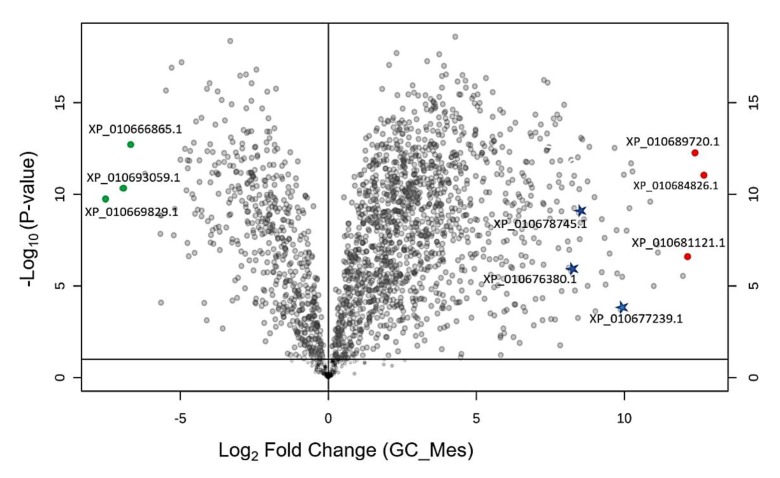
Comparison of differently abundant proteins in mesophyll and guard cells. Volcano plots displaying differentially abundant proteins in mesophyll and guard cell (FDR < 0.05). Three proteins with highest abundance and three proteins with lowest abundance in guard cells (GC) relative to mesophyll are marked in red and green, respectively. GC: guard cell; Mes: mesophyll, C: control conditions, S: saline conditions (300 mM NaCl).

To investigate responses of stomatal to salt stress, we used proteomics to analyse mechanically prepared guard-cell enriched epidermal peels. Isolated GCs-enriched fragments were first examined under the microscope to confirm that no contamination of mesophyll fragments or vascular particles were present. Unlike GCs, epidermal pavement cells are vulnerable and could be destroyed even by manual peeling [16]. Viability test showed that 90% of GCs on epidermis were alive. Typical examples of contaminated GC and purified GC samples have been provided in Supplemental Figures S2 and S3, respectively. To demonstrate the effectiveness of our technique for GC isolation, we first compared proteomic datasets for GC-enriched samples with whole mesophyll samples. At the level of individual proteins, we found that Rubisco (XP_010669829.1) and chlorophyll-related proteins such as chlorophyll a-b binding protein (XP_010666865.1) and photosynthetic NDH (XP_010672059.1) were substantially higher in the mesophyll compared to the GCs (data points indicated in green in Figure 4). These findings are fully consistent with the fact that GCs have functional chloroplasts just as mesophyll cells. However, GCs contain much lower number of chloroplasts per cell and lower amount of Rubisco [17].

**Figure 5:**
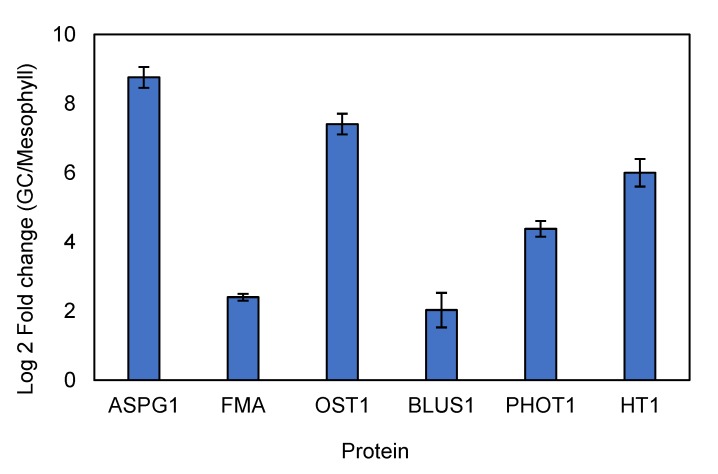
Fold change of selected guard cell-specific proteins in guard cell samples compared to mesophyll. Values are means of 5 replicates for GCs and 4 replicates for mesophyll ± SE. ASPG1: aspartic protease in guard cell 1; FMA: transcription factor; OST1: open stomata-1; BLUS1: blue light signaling 1; PHOT1: Phototropin-1; HT1: high leaf temperature 1.

Interestingly, stress related proteins such as GDSL esterases/lipases (XP_010684826.1), peroxidase P7-like (XP_010689720.1), and hothead-like protein (XP_010681121.1) were more abundant in GC’s (data points indicated in red). Among the stress related proteins, the abundance of abscisic acid receptor PYL2 (XP_010667133.1) and transporter cation/H antiporter 18 (XP_010676380.1) in GCs were 81 and 54 times higher than those expressed in the mesophyll. More importantly, GC-specific proteins such as open stomata1 (OST1), aspartic protease in guard cell 1 (ASPG1), phototropin-1, transcription factor FMA, and serine/threonine-protein kinase BLUS1, high leaf temperature 1 (HT1) were highly accumulated in the GCs (Figure 5), confirming that our guard cell preparation was highly enriched in guard cell proteins. It should be commented that the current procedures for protein extraction or detection are not sufficient for identifying all membrane-bound proteins [18]. Thus, some GCs specific channel proteins such as SLAC1 and KAT1 were not observed in our study. This is in an agreement with previous proteomics reports conducted in Arabidopsis [4,9].

In addition, to identify any major functional differences between the two cell types, enriched GO terms were identified using the web-based AgriGO tool [19]. The complete list of GO categories in mesophyll and GCs are listed in Supplemental Table S1. Generation of precursor metabolites and energy were the most enriched GO categories in GCs, whereas the most significantly-enriched GO categories in the mesophyll were translation and photosynthesis process, suggesting carbon fixation is a major activity in the mesophyll which is consistent with previous findings that the mesophyll proteome was enriched in photosynthesis-related proteins [8].

**Figure 6:**
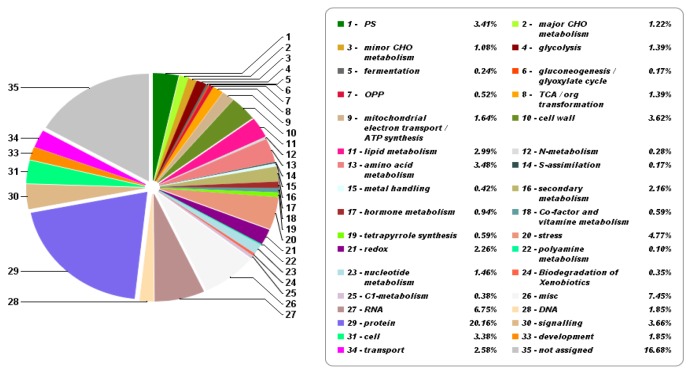
Protein classification of the sugar beet proteome GCs using Mercator 3.6.

To further characterize the GC proteome, we used Mercator4 software in conjunction with MapMan pathway analysis for protein functional classification. Based on the 2,079 GC proteins identified by mass spectrometry, we obtained protein classification for 35 ‘binned’ biological processes (Figure 6).

Protein assignment to the major processes revealed that the largest single group of proteins (~20%) included those involved in protein biogenesis and disposal (e.g., ribosomal subunits, molecular chaperones, and proteasomal subunits). The next most abundant protein categories were identified as “stress” (4.8%), “signaling” (3.7%), “cell wall” (3.6%), “amino acid metabolism” (3.5%), and “photosynthesis” (3.4%). Stress-associated proteins were mainly related to oxidative stress, while signaling proteins and included proteins such as G-proteins, 14-3-3 proteins, MAP kinases, calcium signaling, phosphoinositides, light signaling, signaling in sugar and nutrient physiology, and receptor kinases leucine rich proteins. The complete list of functionally classified GC proteins is provided in the Supplemental Table S2.

### 2.3. Differentially Abundant Proteins in Response to Salt Treatment in Guard Cells (GCs)

**Figure 7:**
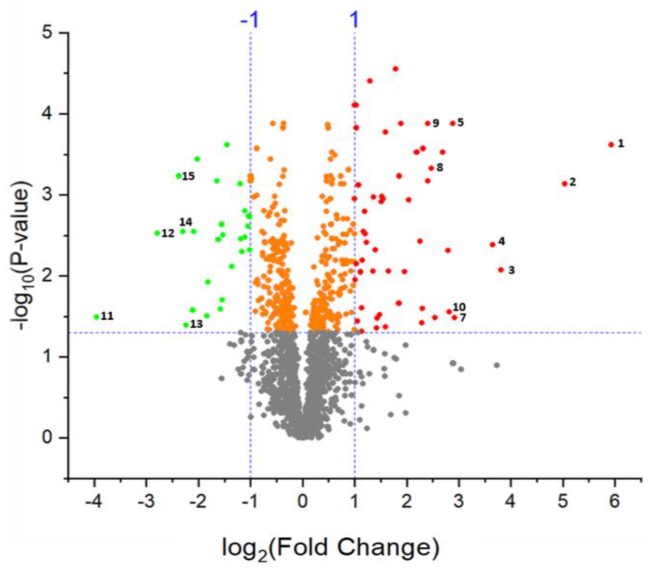
Volcano plot of differentially abundant proteins in the guard cells in response to salt stress.

To identify proteins significantly affected by salt stress we applied a t-test using an FDR-adjusted *p*-value threshold of 0.05 and fold-change cut-off of two. Based on these stringent criteria, changes in abundance of 82 proteins were statistically significant, of which 54 were upregulated and 28 were downregulated by the salt treatment. The 15 most highly modulated proteins by fold-change (10 upregulated and 5 downregulated proteins) are numbered in the volcano plot (Figure 7) and listed in Table 1. The complete list of differentially abundant proteins is provided in Supplemental Table S3.

**Figure 8:**
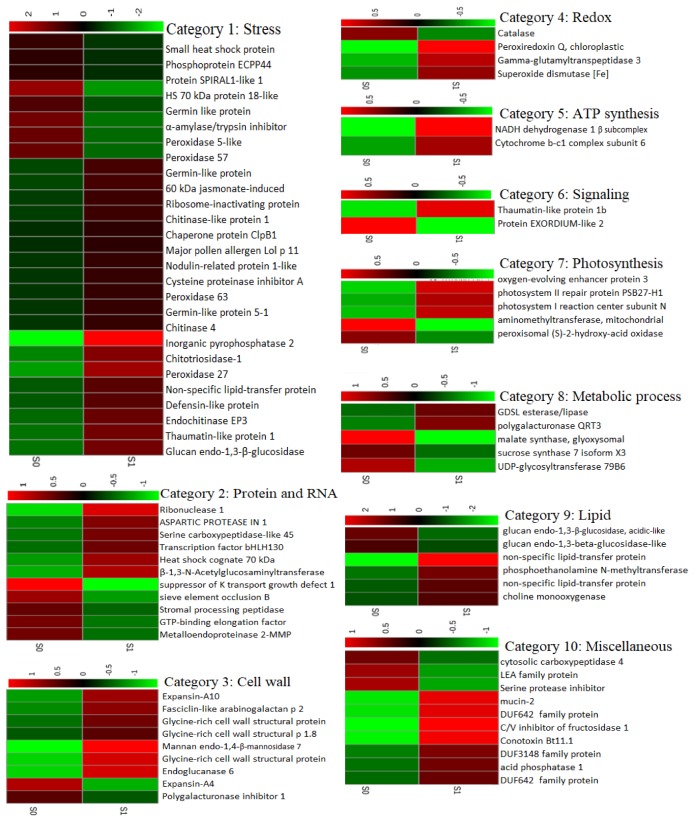
Heat maps (based on Z-scores of the protein abundance measurements) demonstrating abundance patterns of individual proteins within each functional category in sugar beet GCs. The colour intensities shown correspond to the Z-scored LFQ intensities, according to the key shown at the top of each heat map. S0 and S1 denote control and saline treatments, respectively.

We then used MapMan4 to classify the groups of up- and down-regulated proteins according to their major biological functions. These functions, together with the proteins and their associated Z-scored LFQ expression data, are represented as heatmaps in Figure 8. Of the 82 significantly affected proteins, the largest group were assigned to the category “stress”, of which eight were downregulated, and 19 were upregulated under salinity conditions. Several of these proteins are known to be involved in salt and drought stresses (non-specific lipid-transfer protein, inorganic pyrophosphatase 2, 60 kDa jasmonate-induced protein) [20,21,22] and pathogenic stress and other biotic stresses (proteins germin-like) [23].

In the cell wall category, we also observed a mixed pattern of up- and down-regulated proteins. Two isoforms of the expansin family of cell wall proteins were modulated, of which expansin A10 was upregulated, while expansin A4 was downregulated.

Previously it was reported [24] that expansin 2 proteins, which contribute to cell wall extensibility, were upregulated by salt in salt-tolerant genotype but downregulated in salt sensitive one. In addition, enhanced abundance of three members of the family of glycine-rich proteins was also observed.

In the category of protein and RNA biosynthesis, some important proteins with roles in stomatal movement were differentially abundant in response to salt stress. Abundance of aspartic protease in guard cell 1 and transcription factor bHLH130 and heat shock cognate 70 kDa protein increased by 2.5, 2.1, and 2.9-fold, respectively by the salt treatment, whereas other proteins in this category, such as suppressor of K^+^ transport growth defect 1 and protein sieve element occlusion B were markedly decreased by salinity stress. Protein sieve elements have been also found in previous proteomics studies on GC, however, its function in GC is unknown [4].

In plants, reactive oxygen species (ROS) are generated in response to biotic and environmental factors. In guard cells, in addition to external stimuli, endogenous signals such as abscisic acid induce H_2_O_2_ generation and it has been shown that ROS at low levels act as messengers in stomatal movement [25]. In the redox group, we observed that the abundance of peroxiredoxin Q and super oxide dismutase (SOD) enhanced 3.6 and 2.1-fold by salinity while catalase, an antioxidant enzyme with a significant role in protection against oxidative stress, declined 2-fold compared to control.

Another central process in stomatal guard cells is transmembrane ion exchange. This process requires energy provided by photosynthesis and ATP production in mitochondria [26]. In stress condition, more energy is needed to maintain metabolic adjustment [27]. In our study, the abundance of two proteins involved in mitochondrial ATP synthesis was enhanced (3.2 and 2.1-fold) by salinity; no downregulated proteins were found in this group.

Non-specific lipid-transfer proteins are involved in key processes in the plant cell, such as signal transduction, cell wall modification, membrane stabilization, and conferring resistance to salt stress [28]. In our study, two proteins in this group were more abundant in salt-stressed GCs compare to control, including one non-specific lipid-transfer protein that was the most highly increased (61-fold) overall.

### 2.4. Transcription-Level Analysis of Genes Corresponding to Proteins Modulated in Salt-Stressed Guard Cells

**Figure 9:**
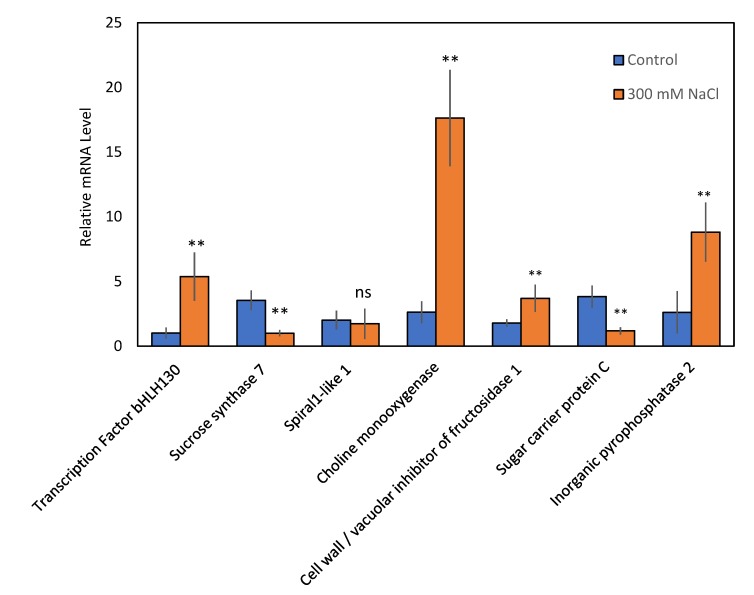
Transcript levels of genes encoding seven altered abundance proteins. Values are means of 3 biological and 3 technical measurements ± SE. Asterisk (*) denotes a significant mRNA difference between control and salt stress for each gene (Student’s t-test, *p* < 0.05).

Salt stress altered the abundance of 82 proteins in sugar beet GCs according to our proteomic analysis. To verify whether alterations in protein abundance are controlled by changes in gene transcription, qRT-PCR was performed for seven genes (Figure 9) known to play an important role in stomatal movement and salt stress responses including transcription factor bHLH130 (XP_010673593.1), sucrose synthase 7 (XP_010675238.1), spiral1-like 1 (XP_010680669.1), choline monooxygenase (XP_010682183.1), cell wall/vacuolar inhibitor of fructosidase 1 (XP_010685378.1), sugar carrier protein C (XP_010686277.1), and inorganic pyrophosphatase 2 (XP_010690044.1).

The mRNA abundance of four genes including transcription factor bHLH130, choline monooxygenase, cell wall/vacuolar inhibitor of fructosidase 1, and inorganic pyrophosphatase 2 in the GCs were all increased by salt stress, while transcript levels of sucrose synthase 7 and sugar carrier protein C were suppressed (Figure 9); these results are fully consistent with the proteomics data. In contrast, expression of spiral1-like 1 at the gene transcript level, which was 2-fold decreased in GCs at the protein level, did not change significantly.

### 2.5. Differentially Abundant Proteins in Mesophyll Under Salinity Treatment

**Figure 10:**
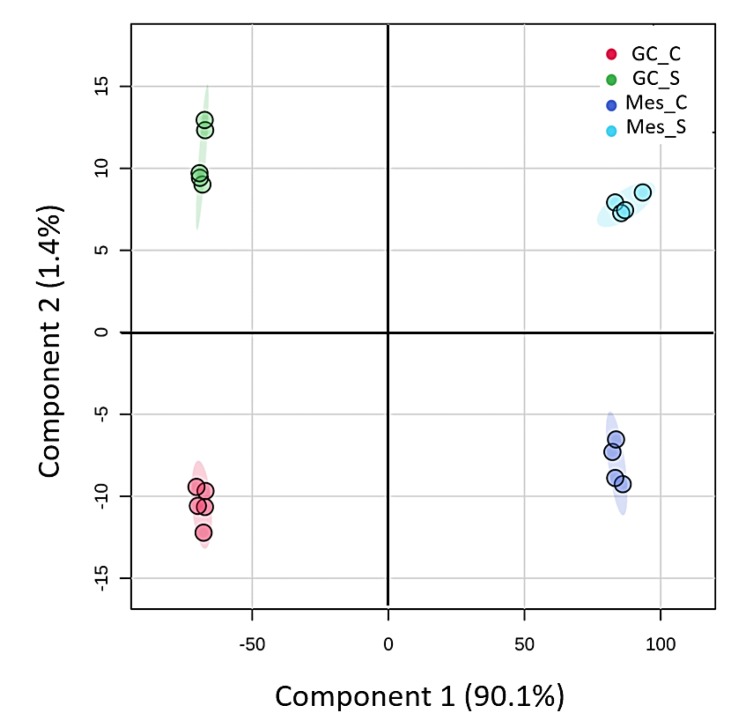
PCA clustering based on mesophyll and guard cell proteome data under control and salt conditions. Proteomics analyses were performed with 4 and 5 biological replicates for mesophyll and guard cells respectively. GC: guard cell; Mes: mesophyll, C: control conditions, S: saline conditions (300 mM NaCl).

Our initial comparison of the mesophyll and GC proteins highlighted differences in composition at level of the functional (GO term) classification and enriched proteins, as would be expected from their fundamentally different roles. This is further supported by results of PCA (plot shown in Figure 10) that illustrates the clear separation between mesophyll and guard cell in PC1, which accounts for ~90% of the variation between samples. To identify whether salinity-induced changes in the GC proteome were also manifest in mesophyll tissue, we compared the two sets of differentially abundant proteins. While fewer proteins were found to be affected in mesophyll tissue based on the same criteria (FDR < 0.05 and FC > 2; Supplemental Table S4), four of the 12 upregulated proteins were also upregulated in GCs. These included non-specific lipid-transfer protein, heat shock cognate 70 kDa protein 2, choline monooxygenase, and phosphoethanolamine N-methyltransferase.

### 2.6. H_2_O_2_ Accumulation in Guard in Response to Salt Stress and Ascorbic Acid

**Figure 11:**
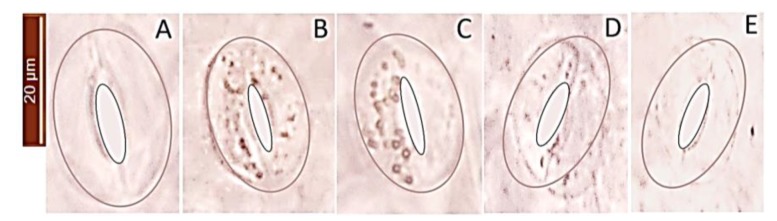
DAB (3,3’-Diaminobenzidine) staining for detection of H_2_O_2_ in guard cell under control (**A**) and 300 mM NaCl (**B–E**). (**B**) control (zero ascorbic acid, AsA); (**C**) 150 mg L^−1^ AsA; (**D**) 300 mg L^−1^ AsA; **E**: 450 mg L^−1^ AsA. Formation of brown precipitates showed the generation of H_2_O_2_ by guard cells. The borders of guard cells and stomatal pores were lined to make it more contrasting with the background. Raw images (without GC outlines) are provided in the Supplemental Figure S4. One (of 15) typical images is shown.

**Figure 12:**
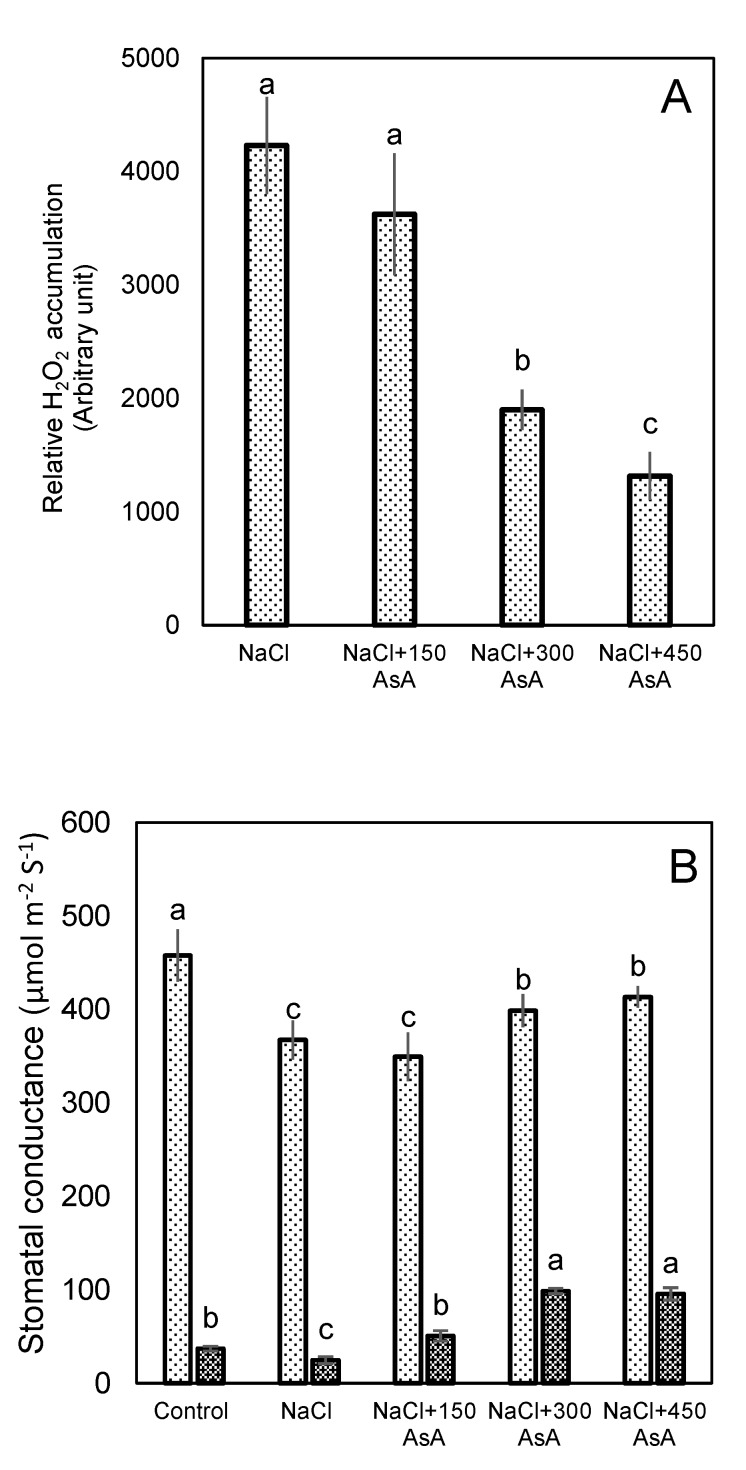
(**A**) Effect of different concentrations of ascorbic acid on H_2_O_2_ of levels in the GCs of sugar beet plants grown under saline conditions, relative to non-stressed controls. Relative signal intensities of DAB staining were quantified using ImageJ software. (**B**) Stomata conductance in non- stressed sugar beet leaves and salt- stressed leaves with or without ascorbic acid treatment. The light bar and dark bar are stomata conductance measurements under natural light conditions and under 30 min-dark conditions, respectively. Values are means of 5 replicates ± SE. Data labeled with different lower-case letters are significantly different at *p* < 0.05.

When plants are exposed to environmental stresses, the level of ROS such as H_2_O_2_ increases and results in oxidative stress and reduction in growth. Consequently, we used DAB (3,3’-Diaminobenzidine) staining to detect H_2_O_2_ in GCs, where formation of brown stain in tissue shows DAB oxidation by H_2_O_2_ (Figure 11 and Figure 12). Our results clearly show elevated H_2_O_2_ levels in salt-stressed GCs compared with controls (Figure 11A,B), which was alleviated by foliar spray of ascorbic acid in a dose-dependent manner (Figure 11C–E). A higher concentration of ascorbic acid was associated with less oxidative status in guard cells. Application of 450 mg L^−1^ ascorbic acid on leaf dramatically reduced the accumulation of H_2_O_2_ in GCs (Figure 11 and Figure 12A). Stomatal conductance measured at natural light conditions in the glasshouse showed that salt-induced reduction in stomatal conductance can be mitigated by application of ascorbic acid. Stomatal conductance measurements in plants after kept under dark conditions for 30 min showed that higher concentrations of ascorbic acid had detrimental effects on stomatal closure. As it can be seen in Figure 12B, stomatal conductance under control conditions was 458 µmol m^−2^ s^−1^ in the light and then reduced to 36 µmol m^−2^ s^−1^ when plants were kept in darkness for 30 min, implying a 92% reduction in stomatal conductance. At the same time, the corresponding values for plants treated with 450 mgL^−1^ of ascorbic acid were 413 and 92 µmol m^−2^ s^−1^ in light and dark, respectively (a 77% reduction). These results indicate that H_2_O_2_ is needed for optimal operation of the stomata, suggesting that tempering with H_2_O_2_ concentration in GCs may result in a lower speed of stomatal closure in response to dark, with the consequences of reducing water use efficiency.

## 3. Discussion

In most proteomics studies, GCs are not analyzed separately but included in the bulk of the leaf samples, thus masking GC-specific mechanisms underlying stomatal operation under stress conditions. To date, a very limited number of studies have used proteomics to analyze GCs specifically [4,8,9,10,29] and none have investigated the specific response of GCs to salt stress. In this study, the efficiency of the GC isolation method was validated with a combination of techniques, including microscopic observation, viability tests, and at the proteome level. Several proteins encoded by GC-specific genes such as ASPG1, OST1, PHOT1, and BLUS1 were markedly higher in GC-enriched samples compared to mesophyll, suggesting that our approach for GC isolation was appropriate for proteomics studies.

### 3.1. Most Abundant Proteins in GCs 

The top protein according to overall abundance (LFQ value) in both control and salt-treated sugar beet GCs was a protein with as-yet uncharacterized function in sugar beet and most plants. However, it is known to be an orthologue of DUF642 protein, encoded by AT5G11420 gene in Arabidopsis. There is evidence that this member of the DUF642 family regulates the activity of pectin methyl esterase [30] which plays a significant role in GCs wall flexibility [31]. DUF642 overexpression increased plant tolerance to pathogenic infection and accelerated plant growth in tomato.

GDSL esterase/lipase a lipolytic enzyme GDSL esterase/lipase along with auxin binding proteins (ABP19) were also among the most highly abundant proteins in sugar beet GCs. The higher abundance of GDSL protein has been reported in response to biotic and abiotic environmental factors [32]. Increased in GDSL esterase/lipases proteins and stomatal apertures were observed in response to low CO_2_ in Arabidopsis [29].

The plant hormone auxin is required for stomatal patterning and development [33] and auxin signaling is dependent on auxin binding proteins. Accumulation of ABP1 protein resulted in higher sensitivity to potassium current in tobacco GCs [34], showing the importance of this protein in GCs performance. Another highly abundant protein in sugar beet GCs was DNA damage-responsive protein (DDR48). This protein is reported to be involved in mitigating oxidative stress and resistance against fungal attack [35], suggesting the constitutive accumulation of those proteins can confer augmented tolerance to GCs when exposed to salt stress.

### 3.2. Stress Related Proteins Differentially Abundant Proteins in GCs Under Salinity Treatment

A high proportion of differentially abundant proteins in the GCs of salt-treated plants were related to various types of stresses including salinity, drought, oxidative, cold, and biotic stresses. Three enzymes that were among the most highly up-regulated proteins under salinity stress, namely pyrophosphatase (PPase), chitotriosidase-1, and peroxidase, are well documented for their roles in conferring salt tolerance in multiple species, especially halophytes [36,37]. Pyrophosphatase, which was upregulated 45-fold by salt stress, is a proton pump capable of energizing the vacuolar membrane for generating electric potential and transporting H^+^ across the membrane [38] and thus is crucial for ion transport in the GCs. Overexpression of halophytic PPase genes in glycophytes resulted in a higher accumulation of Na^+^ by plants [36], while PPase loss-of-function mutants showed high susceptibility to water deficiency. Stomata malfunction is the main interpretation for the drought sensitivity of mutant plants with a low abundance of this enzyme, where stomatal closure is delayed after ABA treatments in H^+^-PPase mutants.

Nodulin-related protein 1 was 2-fold higher in GCs of salt-treated plants compared with controls. Previously, it was shown that this gene was induced by the cold stress and suppressed by the heat stress in Arabidopsis, and changes in this protein are associated with increased tolerance to various types of biotic and abiotic stress [39].

Multiple non-specific lipid-transfer proteins (LTPs) were highly induced by salt in the GCs. LTPs [40] mediate cell signaling in response to environmental factors and have been associated with adaptation of plants to stresses and stimuli [28]. XP_010683515.1 and XP_010690794.1 (upregulated by 61- and 4-fold, respectively) are homologous of LTP4 in Arabidopsis which is involved in response to salt and dehydration [22,41,42]. Induction of this gene has been reported in foxtail millet in response to salt stress and ABA treatment. Furthermore, overexpression of LTPs using gene editing increased plant tolerance to desiccation and osmotic stress, by the accumulation of soluble sugars and proline. Analysis of a specific LTP gene promotor region (isolated from *Setaria italica*) identified two dehydration-responsive elements (DRE) proteins that could bind to abscisic acid–responsive transcription factor. Thus, LTP is likely a downstream gene of this ABA-responsive transcription factor [42].

A wide variety of chitinases have been identified in plants (class I-class V). While plants do not contain chitin compounds, chitinase enzymes are either expressed constitutively or induced in response to biotic and abiotic stresses [43]. In our study, four chitinase isoforms were found among upregulated proteins. Levels of chitotriosidase-1 increased 10.3-fold in salt-stressed GCs compared to the control. This protein is homologous of chitinase V in Arabidopsis which has a significant role in salt stress in addition to defense against pathogenic attack [43], and loss of function of the gene encoding chitinase-like protein results in overaccumulation of Na^+^ in the cytosol [44]. In stomata, it has been reported that chitosan, which can be produced by chitinase, mediates stomatal narrowing and it may be advantageous for both the salt stress responses and plant defense [45,46].

Several proteins involved in biotic stress (such as the disease-related protein 60 kDa jasmonate-induced protein) and defense response to fungus (such as defensin-like protein) were also upregulated in response to salt stress. In addition, other proteins related to biotic and abiotic stress, such as stress protein DDR48, various isoform of peroxidase and L-ascorbate oxidase were constitutively accumulated in the sugar beet GCs. This data supports a role for these proteins in the inherent tolerance of sugar beet GCs to tolerate multiple stresses, which can then be further enhanced by upregulation of other proteins in response to salinity.

### 3.3. Redox-Related Proteins

Salt stress causes an overproduction of ROS, which can damage DNA, proteins, and carbohydrates. In order to prevent accumulation of ROS to toxic levels, enzymatic and non-enzymatic antioxidants are usually generated to scavenge the surplus of ROS. In the current study, we found an increase in two peroxidase isoforms (POD27 and POD63) and enhanced levels of superoxide dismutase (SOD), while catalase and two other POD isoforms showed lower abundances in GCs of salt-treated plants. Additionally, L-ascorbate oxidase was among the most highly abundant proteins in sugar beet GCs, under both control and salt treatment. This protein is plant specific and involved in cellular redox regulation through decreasing oxygen content (thus limiting ROS generation) and oxidizing AsA which is a crucial antioxidant [47]. The essentiality of stress-induced increase in AsA was tested in direct experiments by studying effects of exogenous supplied ascorbic acid onto redox status of guard cells. A high concentration of H_2_O_2_ was detected in GCs due to salt stress (Figure 11 and Figure 12); this stress-induced elevation was mitigated by the application of ascorbic acid. Concurrently, AsA-treated plants had a higher degree of stomatal aperture opening, and consequently higher stomatal conductance, under salt stress. Interestingly, when salt-grown plants that had been sprayed with ascorbic acid were exposed to the darkness, they showed a delay in the stomatal closure. These findings are consistent with the role of H_2_O_2_ as a modulator of stomata functioning [48].

### 3.4. Photosynthesis and ATP Synthesis

It has been documented that GC photosynthesis is necessary for stomatal opening [26] and closure [49]. We found that three proteins related to photosynthesis and two related to ATP synthesis were upregulated by salt in sugar beet GCs, including oxygen-evolving enhancer protein 3 and photosystem I reaction center subunit N. Another photosynthesis-related protein highly upregulated under saline conditions was photosystem II repair protein PSB27-H1, which is involved in the stability of photosynthesis systems and repairing PSII after damage. Moreover, peroxiredoxin Q, which is involved in redox homeostasis in the chloroplast, was also induced by salt showing the ability of sugar beet GCs in maintaining photosynthesis under saline conditions. Stomata rely on the production of ATP in the GCs for their movements. Under salinity stress, more energy is required for osmotic adjustment and dealing with toxin ions [50]. Therefore, it is not surprising that proteins associated with ATP synthesis were upregulated in the GCs by salinity treatment.

### 3.5. Protein and RNA Biosynthesis

Transcription factor bHLH130, a member of the basic helix-loop-helix (bHLH) family and homologous with the Arabidopsis ABA-responsive kinase substrate 1 gene, was upregulated by salt treatment. Under non-stress conditions this gene promotes stomatal opening by the transcription of genes encoding potassium channels [51]. However, in the presence of ABA, its activity is suppressed through phosphorylation of bHLH, causing a reduction in light-induced stomatal opening [51].

The GC-specific protein aspartic protease in guard cell 1 was also upregulated in response to salinity. Following previous results, this protein was among the most highly differentially abundant compared with mesophyll tissue [52]. Overexpression of this protein was previously found to be accompanied by drought avoidance by higher sensitivity to ABA in the stomatal closure and lower the rate of transpirational water loss [53].

### 3.6. Cell Wall

The function of the GC cell walls is crucial in stomatal dynamics and differentiation [54]. The cell wall of GCs is required to be stiff and sufficiently strong to withstand high turgor pressure when stomata are opening and be flexible to reverse this when stomata are closing. Cell wall modifications, such as loosening, is important in plants under drought and salt stress conditions [55] and may affect stomatal movements. As such, mannan endo-1,4-beta-mannosidase 7, which is known for loosening the cell wall, was strongly upregulated (7.5-fold) in sugar beet GCs. Some structural proteins which allow flexibility of cell wall such as glycine-rich proteins (GRP) were also highly abundant in the GCs. It has been reported that some isoforms of this family (GRP1.8) have been digested by collagenase, suggesting that they are structurally similar to collagen in animals and allow cell extensibility [56].

### 3.7. Lipid Metabolism

In the category of lipid metabolism, two key enzymes phosphoethanolamine N-methyltransferase and choline monooxygenase, which are involved in choline and glycine betaine biosynthesis, were highly upregulated (6.6- and 3.7-fold). Glycine betaine is a water-soluble molecule involved in abiotic stresses by mitigating ROS impacts [57] and contributing to osmotic adjustment as a compatible solute [58]. Successful germination of sugar beet seeds under high salt levels was attributed to the high accumulation of glycine betaine in the seed [59].

### 3.8. Proteins Downregulated in Sugar Beet GCs in Response to Salinity

About one-third of total differentially abundant proteins in response to salt stress were downregulated in sugar beet GCs. Previous studies have documented the down-regulation of specific proteins in adaptation to salinity, including the microtubule organizer spiral1 protein which is degraded in order to dissemble microtubules and to accelerate the plant response to salinity [60]. In our study, this protein was down-regulated 2-fold in GCs under salinity stress. Generally, salt stress causes spiral1 proteolysis and lower abundance of this protein is required for managing salt stress.

The abundance of protein suppressor of K^+^ transport growth defect 1 declined by salinity in our study. It has been reported that decreased expression of this gene in Arabidopsis resulted in reduced salinity tolerance through an imbalanced Na^+^/K^+^ ratio [61].

Sugar carrier protein C was suppressed by salinity in the GCs in this study. In *Pisum sativum,* it has been reported that sugars act as the osmoticum and support the stomatal opening [62]. Downregulation of this protein may associate with less turgor pressure in the GCs and less stomatal aperture, which is advantageous for plants under salt stress.

## 4. Materials and Methods

### 4.1. Growth conditions and physiological Parameters

Seeds of sugar beet (*Beta vulgaris ssp. vulgaris*) were obtained from Leibniz Institute of Plant Genetics and Crop Plant Research (IPK) (gbis.ipk-gatersleben.de), Germany. Plants were grown in 20 cm diameter pots (six plants per pot) filled with potting mix containing 90% composted pine bark; 5% coco peat; 5% coarse sand dolomite (6 kg/m^3^); gypsum (1 kg/m^3^); ferrous sulphate (1.5 kg/m^3^); slow-released fertilizer and Osmoform Pre-mix (1.25 kg/m^3^) Scotts Pro (3 kg/m^3^) at the glasshouse facilities at the University of Tasmania in Hobart, Australia. The mean day/night temperatures were 24 °C/16 °C and relative humidity was 70%. Salt stress was imposed 3 weeks after planting by adding 300 mM NaCl to the irrigation water and lasted for another 3 weeks. For CO_2_ assimilation rate and stomatal conductance (gs) measurement a Li-Cor 6400 gas analyzer system (Lincoln, NE, USA) was used (light intensity:1500 µmol m^−2^ S^−1^, CO_2_ concentration: 400 μmol mol^−1^, atmospheric VDP: 75% RH and leaf temperature: 20 °C). A portable fluorometer (Heinz Walz GmbH, Effeltrich, Germany) was used for the maximal photochemical efficiency of PSII (Fv/Fm) measurements. Youngest fully expanded leaves were used for all measurements.

### 4.2. In Situ Detection of H_2_O_2_ in Guard Cells and Ascorbic Acid Treatments

Hydrogen peroxide in guard cells was detected using 3,3’-Diaminobenzidine (DAB) staining. For this purpose, leaves from control and salt treated plants were incubated in DAB solution (1 mg mL^−1^, DAB, and 10 mM Na_2_HPO_4_. Vacuum-infiltrating was applied for 5 min in the dark. Then samples, were kept on the shaker overnight at 80 rpm shaking speed (room temperature and darkness). For de-staining, the samples were washed with ethanol: glycerol: acetic acid (3:1:1) solution two times before visualizing in light microscopy. DAB staining processes were repeated for the salt-grown leaves treated with different concentrations of ascorbic acid. For ascorbic acid experiment concentrations of 150, 300, and 450 mg L^−1^ ascorbic acid were sprayed on the leaf 3 times a day for three consecutive day, then the stomatal conductance were measured when plants were in the normal light in the glasshouse, then plants moved to a dark place and kept for 30 min then the stomatal conductance were measured again.

### 4.3. GC and Mesophyll Preparation

GC-enriched epidermal peels were prepared from fully expanded leaves of well-watered 3-4-week-old sugar beet. The leaves were ground in the Grindomix blender (GRINDOMIX LENDER GM 200, Retsch, Germany) with 200 mL water and 100 mL crushed ice by 3 pulses of 20 seconds at maximum speed. The leaf mixture was passed through a 210 µm Nylon mesh (ELKO, Filtering co. USA) and rinsed with ice-cold distilled water and small dark green tissue fragments were removed using clean forceps (three times). Samples were snap-frozen in liquid nitrogen and were kept in −80 °C freezer until used for protein extraction. Isolated GCs-enriched fragments were examined under the microscope to confirm that no contamination of mesophyll fragments or vascular particles (green tissue) were present. Separation of the mesophyll tissue was performed mechanically. Fully expanded leaves were excised and adaxial (upper) side of leaf was attached with double-sided tape strips that had been fixed on the laboratory bench. The epidermis of abaxial sides were peeled off using a forceps and mesophyll tissue were cut using a surgical scalper and collected in aluminum foil before snap freezing in liquid nitrogen.

### 4.4. Protein Extraction

GC samples from the control and salt treatments were ground to a fine powder in liquid nitrogen (five biological replicates per treatment). Then, 10% trichloroacetic acid (TCA) in pre-chilled acetone containing 0.07% (*v*/*v*) 2-mercaptoethanol was used to precipitate total proteins. The homogenate was kept at −20 °C overnight. Then the samples were centrifuged at 20,000× *g* at 4 °C for 20 min and the pellets were washed in 100% pre-chilled acetone containing 0.07% (*v*/*v*) 2-mercaptoethanol. This wash step was repeated until all visible traces of chlorophyll were removed. After a final wash in 80% prechilled acetone containing 0.07% (*v*/*v*) 2-mercaptoethanol protein pellets were centrifuged then air-dried at room temperature. Proteins were then solubilized in 1ml solubilization buffer (7 M urea, 2 M thiourea, and 40 mM Tris) at 20 °C for 1 h then clarified by centrifugation. Protein extracts were precipitated with 4 volumes of acetone and resuspended in 50 μL of solubilization buffer containing 7 M urea, 2 M thiourea, 40 mM Tris. Protein concentrations were measured using Pierce 660 nm Protein Assay Reagent. Protein concentrations were from 530 μg/mL to 750 μg/mL in different treatments and biological replications. Samples with higher concentrations of protein were diluted to obtain a constant amount of proteins in all samples. All centrifugation steps were at 20,000× *g* at 4 °C for 30 min.

### 4.5. Enzymatic Digestion and Off-Line Desalting

For each sample, 100 μg of precipitated proteins from GCs were reduced, alkylated, and digested with trypsin at a ratio of 1:50 and kept for 5 h at 37 °C. Digested samples were desalted using Millipore ZipTip C18 according to manufacturer’s instructions and dried peptides were reconstituted in 12 μL HPLC loading buffer (2% acetonitrile and 0.05% TFA in water), then stored at −80 °C until LC–MS/MS analysis.

### 4.6. Protein Identification by NanoLC–MS/MS

Peptides were first concentrated on a 20 mm × 75 μm PepMap 100 trapping column (3 μm C18) for 5 minutes then separated using a 250 mm × 75 μm PepMap 100 RSLC column (2 μm C18) at a flow rate of 300 nL/min and held at 45 °C. A 90-minute gradient from 98% mobile phase A (0.1% formic acid in water) to 50% mobile phase B (0.08% formic acid in 80% acetonitrile and 20% water) comprised the following steps: 2–10% B over 12 min, 10–25% B over 48 min, 25–45% B over 10 min, holding at 95% B for 5 min then re-equilibration in 2% B for 15 min. The nano HPLC system was coupled to a Q-Exactive HF mass spectrometer equipped with nanospray Flex ion source (Thermo Fisher Scientific, MA, USA) and controlled using Xcalibur 4.1 software. Spray voltage was set to 2.0 kV, S-lens RF level to 50 and heated capillary set at 250 °C. MS scans were acquired from 370–1500 m/z at 60,000 resolution, with an AGC target of 3 × 10e6 and a maximum fill time of 100 ms. Fragment ion scans were acquired at 15,000 resolution (scan range 200–2000 *m*/*z*), with an AGC target of 2 × 10e5 and a maximum fill time of 28 ms. An isolation width of 1.4 *m*/*z* was used, and normalized collision energy for HCD set to 27eV. MS/MS spectra were acquired in data-dependent mode using a Top15 method with 30-second dynamic exclusion of fragmented peptides.

### 4.7. Database Searching and Protein Quantitation

Raw data files were imported into MaxQuant version 1.6.5.0 (http://maxquant.org/) for label-free quantitative comparison between control and saline GC samples and mesophyll samples. GC samples were injected in duplicate and LFQ data combined at the sample level using the same Experiment identifiers in the MaxQuant experimental design table. Mesophyll samples were injected once only and defined as a separate parameter group, with the MaxLFQ algorithm applied separately to this sample group. MS/MS spectra were searched using the Andromeda search engine against protein databases downloaded from NCBI for *Beta vulgaris*. Default settings for protein identification by Orbitrap MS/MS were used, with the match-between-runs match time window set to 0.7 min, including a maximum of two missed cleavages, mass error tolerances of 20 ppm, then 4.5 ppm for initial and main peptide searches, respectively, 0.5 Da tolerance for fragment ions, carbamidomethyl modification of cysteine and variable methionine oxidation. A false discovery rate of 0.01 was used for both peptide-spectrum matches and protein identification. The MaxQuant peptides.txt and proteinGroups.txt output files are presented in Supplemental Tables S5 and S6. The mass spectrometry proteomics data have been deposited to the ProteomeXchange Consortium via the PRIDE partner repository with the dataset identifier PXD017583.

### 4.8. Determination of Relative Protein Abundance and Statistical Analysis

We utilized MaxLFQ, the MaxQuant algorithm, for peptide intensity determination and normalization [63] using pair-wise comparison of unique and razor peptide intensities and a minimum ratio count of 2. The proteinGroups output files generated by MaxQuant analysis were processed as follows: The normalized label-free quantification (LFQ) intensity values, MS/MS counts, and the numbers of razor and unique peptides for each of the identified proteins were imported into Perseus software version 1.5.031 (http://perseus-framework.org/). Protein groups identified either as potential contaminants (prefixed with CON), identified by modified site only, by reverse database matching or on the basis of a single matching peptide, were removed. LFQ intensity values were then log2−transformed and then a filter applied to include only proteins detected in a minimum of 70% of the samples. Missing values were replaced with random intensity values for low-abundance proteins based on a normal distribution of protein abundances using default MaxQuant parameters. A Student’s T-test was run on log2− transformed values to identify the differentially abundant proteins between non stressed and salt-stressed samples. Proteins with an FDR < 0.05 were considered to have statistically significant changes in abundance.

### 4.9. Real-Time Quantitative Reverse Transcription PCR Analysis

To validate the abundance of some proteins at the transcription level, mRNA levels of seven differentially abundant proteins were carried out. The primers were designed for amplification of differentially expressed genes using primer-blast software (https://www.ncbi.nlm.nih.gov/tools/primer-blast/). Actin was used as a reference gene [64]. The list of gene-specific primer sequences is provided in the Supplemental Table S7. Total RNAs were extracted from GC samples taken from control and salt-treated plants using QIAGEN Kit and TransScript RT-PCR Kit was used for reverse transcription for cDNA synthesis. The reaction conditions for RT- PCR were as follows: 94 °C for 3 min for initial denaturation, followed by 35 cycles of 94 °C (30 s), 55 °C (30 s) and 72 °C (30 s) with final extension of 5 min at 72 °C. Quantifications for each gene was normalized to actin DNA.

### 4.10. Annotation and Gene Ontology (GO) Enrichment Analysis

The Basic Local Alignment Search Tool (BLAST) was used for obtaining the protein sequences of each accession number in GCs proteome. Amino acid sequences of all GCs proteins used as input in most recent version of MapMan4 tool, using Mercator4 software (https://plabipd.de/portal/mercator4) [65] to obtain functional classification of GCs proteins. MapMan framework like The Kyoto Encyclopedia of Genes and Genomes (KEGG) ontology covers various aspects of biology based on massive databases. Both frameworks use the same structure of protein function terms [65].

Gene ontology (GO) term annotation and GO enrichment analysis for GC proteins and differentially abundant proteins were determined using agriGO ver.2 [19]. Heatmap and PCA plots were drawn using MetaboAnalyst software (https://www.metaboanalyst.ca) and volcano plots were obtained from Origin software (https://www.originlab.com).

### 4.11. Statistical Analyses

Statistical analyses on physiological data were performed with IBM SPSS Statistics software, version 26 (IBM Corp., Armonk, NY, USA). Statistical significance was determined by one-way ANOVA analysis based on Duncan’s test. The differences between means were considered statistically significant as *p*-values were less than 5%.

## 5. Conclusions

In this study, we have used proteomics to investigate the effects of salinity on guard cells for the first time, using a halophilic plant species with potential to be cultivated under saline conditions. The abundances of proteins related to lipid metabolism, cell wall modification, ATP biosynthesis, and signaling were modified in the GCs of sugar beet plants in response to salt stress. A high proportion of differentially abundant proteins were involved in various types of stresses including salt, drought, oxidative, cold, and biotic stresses. Moreover, some proteins involved in mitigation of oxidative stress were presented at elevated levels under non-stress conditions suggesting the constitutive accumulation of those proteins in sugar beet GCs that can confer augmented tolerance to GCs when exposed to salt stress. Among those proteins that were most highly abundant and/or accumulated by salinity treatment were several proteins currently of unknown function. These proteins represent good candidates for further research into their functions in GCs using molecular and genetic approaches.

## Figures and Tables

**Table 1:** The 15 most highly modulated proteins (10 upregulated and 5 downregulated proteins).

No.	Accession Number	Log2 LFQ Intensity (Control)	Log2 LFQ Intensity (Salt Stress)	Unique Peptides	Fold Change	t-Test q Value	Annotation
1	XP_010683515.1	23.1	29.0	4	60.9	2.4 × 10^−4^	non-specific lipid-transfer protein
2	XP_010690044.1	23.1	28.6	8	45.0	7.3 × 10^−4^	inorganic pyrophosphatase 2
3	XP_010680759.1	23.9	27.3	7	10.3	8.5 × 10^−3^	chitotriosidase-1
4	XP_010679746.1	25.2	28.5	14	10.2	4.2 × 10^−3^	peroxidase 27
5	XP_010679767.1	26.0	28.9	14	7.5	1.3 × 10^−4^	mannan endo-1,4-beta-mannosidase 7
6	XP_010686587.1	28.0	30.7	24	6.6	3.0 × 10^−4^	phosphoethanolamine N-methyltransferase
7	XP_010676511.1	22.6	25.1	4	5.7	3.3 × 10^−2^	thaumatin-like protein 1
8	XP_010679501.1	26.9	29.4	2	5.3	4.7 × 10^−4^	glycine-rich cell wall structural protein
9	KMT11925.1	27.0	29.4	16	5.3	1.3 × 10^−4^	endoglucanase 6
10	XP_010687451.1	23.0	25.4	2	5.2	2.8 × 10^−2^	endochitinase EP3
11	XP_010678753.1	26.5	23.1	3	−10.25	3.2 × 10^−2^	heat shock 70 kDa protein 18-like
12	XP_010677928.1	26.5	23.8	8	−6.51	3.0 × 10^−3^	malate synthase, glyoxysomal
13	XP_010686038.1	27.1	24.6	2	-5.72	3.1 × 10^−2^	protein suppressor of K^+^ transport growth defect1
14	KMT14555.1	30.2	27.8	6	-5.39	2.8 × 10^−3^	glucan endo-1,3-beta-glucosidase, acidic-like
15	XP_010676519.1	30.3	27.8	8	-5.32	5.9 × 10^−4^	alpha-amylase/trypsin inhibitor

## Supplementary Materials

Supplementary materials can be found at https://www.mdpi.com/1422-0067/21/7/2331/s1.

## Abbreviations

DAB	3,3’-Diaminobenzidine
GC	Guard cell
GO	Gene Ontology
Mes	Mesophyll
